# Myosin IXa Binds AMPAR and Regulates Synaptic Structure, LTP, and Cognitive Function

**DOI:** 10.3389/fnmol.2016.00001

**Published:** 2016-01-20

**Authors:** Alessandra Folci, Luca Murru, Elena Vezzoli, Luisa Ponzoni, Laura Gerosa, Edoardo Moretto, Fabiana Longo, Jonathan Zapata, Daniela Braida, Francesco Pistillo, Martin Bähler, Maura Francolini, Mariaelvina Sala, Silvia Bassani

**Affiliations:** ^1^CNR Institute of NeuroscienceMilano, Italy; ^2^Institute of Biophysics, Medical University of GrazGraz, Austria; ^3^Department of Medical Biotechnologies and Translational Medicine (BIOMETRA), Università degli Studi di MilanoMilano, Italy; ^4^Department of Pharmacological and Biomolecular Sciences, Università degli Studi di MilanoMilano, Italy; ^5^Institute of Molecular Cell Biology, Westfälische Wilhelms-Universität MünsterMünster, Germany

**Keywords:** Myosin IXa, hippocampal synapses, PSD, AMPAR, LTP

## Abstract

Myosin IXa (Myo9a) is a motor protein that is highly expressed in the brain. However, the role of Myo9a in neurons remains unknown. Here, we investigated Myo9a function in hippocampal synapses. In rat hippocampal neurons, Myo9a localizes to the postsynaptic density (PSD) and binds the alpha-amino-3-hydroxy-5-methyl-4-isoxazole propionic acid receptor (AMPAR) GluA2 subunit. Myo9a^+/-^ mice displayed a thicker PSD and increased levels of PSD95 and surface AMPAR expression. Furthermore, synaptic transmission, long-term potentiation (LTP) and cognitive functions were impaired in Myo9a^+/-^ mice. Together, these results support a key role for Myo9a in controlling the molecular structure and function of hippocampal synapses.

## Introduction

Myosin IXa (Myo9a, previously also called myr7) is an actin-dependent motor protein of the unconventional myosin IX class. Myo9a is expressed in several tissues and is enriched in the brain and testes (Chieregatti et al., 1998; Gorman et al., 1999). In the brain, Myo9a is detectable in all regions, particularly in the hippocampus, cortex, and cerebellum, during both development and in adulthood (Chieregatti et al., 1998).

Class IX myosins share a common structure: an N-terminal extension preceding a myosin head, a head (motor) domain, a neck with IQ motifs that bind light chains and a C-terminal tail containing a Rho-GTPase activating protein (RhoGAP) domain. The RhoGAP domain enables class IX myosins to inactivate small GTPases of the Rho family (Reinhard et al., 1995).

Myo9a knockout mice develop hydrocephalus and kidney dysfunction, highlighting the importance of Myo9a in epithelial cell morphology and differentiation (Abouhamed et al., 2009; Thelen et al., 2015). However, the role of Myo9a in neurons has not yet been investigated.

Here, we found that Myo9a is expressed in excitatory synapses and interacts with the GluA2 subunit of the alpha-amino-3-hydroxy-5-methyl-4-isoxazole propionic acid receptor (AMPAR) in rat hippocampal neurons. AMPARs mediate fast excitatory transmission in the brain, and their abundance and distribution at synapses are crucial determinants of synapse strength and plasticity (Bredt and Nicoll, 2003; Kessels and Malinow, 2009). We therefore postulated a role for Myo9a in the synapse and investigated its involvement in hippocampal long-term potentiation (LTP) and cognitive behavior. Interestingly, mice expressing reduced levels of Myo9a (Myo9a^+/-^ mice) displayed a thicker postsynaptic density (PSD) and increased surface AMPAR levels and showed impaired synaptic plasticity and cognitive defects.

## Materials and Methods

### Animals

Myosin IXa (Myo9a) knockout mice have been described previously in Abouhamed et al. (2009). Littermate male Myo9a^+/+^ (WT) and Myo9a^+/-^ mice were used in accordance with national and European (86/609/EEC) guidelines.

Rats for *in vitro* experiments were obtained from Charles River, Italy, and were sacrificed in accordance with European Communities Council Directive 86/809/EEC. All efforts were made to minimize the number of animals used (reported in section “Statistical Analysis”) and their suffering. *Age of mice used in the experiments*. Mice at 4 weeks of age were used for Golgi staining, electron microscopy (EM), biochemical, and crosslinking assays. For electrophysiological and behavioral studies, we used animals aged 4–6 weeks (for miniature excitatory postsynaptic currents, mEPSCs, and paired pulse), 2–4 months (for LTP) or 2–3 months (for behavior).

### cDNA Constructs

Sequences corresponding to amino acids 2–130, 2135–2338, and 2330–2626 from rat Myo9a (NCBI accession number: AJ001713) were cloned into a pGEX-4T1 vector to obtain the GST N-terminal, GST Rho-GAP, and GST C-terminal. GluA1 (from rat, NCBI accession number: X17184) and GluA2 (from rat, NCBI accession number: M85035) in pGW1 vector were gifts from Dr. M. Passafaro. Sequence corresponding to the GluA2 intracellular C-terminal tail (amino acids 834–883) was cloned into a pGEX-4T1 vector to obtain the GST GluA2 C-term.

### Neuronal Cultures and Biochemical Assays

Dissociated hippocampal neurons were prepared from rat embryos of either sex at embryonic day (E)18 as described in Bassani et al. (2012). Neurons were plated in 6-well plates on coverslips coated with poly-D-lysine (Sigma) at a density of 300.000/well.

#### Immunopurification Experiments

Immunopurification experiments were performed as described in Bassani et al. (2012). Briefly, neurons at days *in vitro* (DIV)18 were lysed in 50 mM Tris-HCl, 150 mM NaCl, 1 mM EDTA, 1% Triton X-100 (v/v), 1% saponin (w/v) and a protease inhibitor cocktail (Sigma). The soluble extracts of neurons were loaded into a column of resin (cyanogen bromide-Sepharose 4B resin) bound to anti-GluA2/3 antibodies and incubated overnight at 4°C. The column was subsequently washed with PBS (0.1% Triton X-100). AMPAR binding proteins were eluted with glycine (0.2 M, pH 2.2) and separated by SDS-PAGE.

#### Immunoprecipitation Experiments

Mouse hippocampal homogenates were prepared in buffer containing 150 mM NaCl, 1 mM EDTA, 50 mM Tri-HCL, 0.5% NP-40, 0.5% Triton X-100, proteases inhibitors, pH 7.4. Homogenates were incubated overnight at 4°C with anti-Myo9a antibodies (Tü 78, 10 μg/ml; Hanley et al., 2010) or rabbit IgG (control, 10 μg/ml). Protein A agarose beads (Invitrogen) were added, and incubation continued for 2 h. The beads were collected via centrifugation and were washed with lysis buffer (three times) and PBS plus protease inhibitors (three times), re-suspended in sample buffer and boiled for 5 min for SDS-PAGE.

#### PSD Fractionation

The PSD fraction was prepared from rat brains and subjected to detergent extraction as described by Wyszynski et al. (1998). PSD fractions purified by sucrose sedimentation were extracted with Triton X-100 once (PSD I), twice (PSD II), or with Triton X-100 followed by sarkosyl (PSD III). Samples were separated using SDS-PAGE and visualized using immunoblotting.

### Pull-Down Assays in Heterologous Cells

HEK293 cells were maintained in DMEM (Life Technologies) supplemented with 10% FBS, 1% GlutaMAX, 1% penicillin, and 1% streptomycin. HEK293 cells at 50–70% confluence (24 h after plating onto 6-well plates) were transiently transfected with cDNA constructs using a JetPEI Transfection Kit (Polyplus Transfection) according to the manufacturer’s instructions. The transfected cells were grown for 24–48 h, washed in PBS and solubilized in lysis buffer (PBS, 1% Triton X-100, 1 mM EDTA, protease inhibitor cocktail, pH 7.4). GST fusion proteins were prepared in *Escherichia coli* strain BL21 and purified according to standard procedures. The lysates were incubated overnight at 4°C with GST fusion protein immobilized on glutathione-Sepharose 4B beads (GE Healthcare), washed three times in lysis buffer and resuspended in SDS sample buffer. Samples were separated by SDS-PAGE followed by western blotting with appropriate antibodies.

The GST GluA2 C-term was expressed in *E. coli* BL21 cells and purified with the glutathione-Sepharose 4B resin. GST GluA2 C-term bound to resin was resuspended in PBS and was incubated with thrombin (Sigma, 10 U/mg of fusion protein) overnight at 4°C to release GluA2 C-termini (GluA2 C-term) in solution. Protease cleavage was stopped with 1 mM PMSF, and the solution containing GluA2 C-term was subsequently incubated with glutathione Sepharose 4B-coupled GST-fusion proteins (GST and GST C-term from Myo9a).

### BS^3^ Crosslinking

The experiments were carried out according to Boudreau et al. (2012). Briefly, mice were sacrificed, and brain slices of 400 μm thickness were cut using a vibratome and rapidly put in ice-cold artificial Cerebral Spinal Fluid (aCSF). Cell membrane impermeable BS^3^ crosslinker (PierceNet) was prepared as a 52 mM stock in 5 mM sodium citrate buffer pH 5. The slices were then put into a 12-wells plate with 1 ml of ice-cold aCSF and BS^3^ added to a final concentration of 2 mM. The plate was incubated for 30 min at 4°C with gentle agitation. Glycine was added to a final concentration of 100 mM and incubated for 10 min at 4°C with gentle agitation to quench the reaction. The slices were then collected and lysed with mechanical homogenization in lysis buffer (50 mM Tris, 150 mM NaCl, 1 mM EDTA, 1% SDS, pH 7.4). The lysates were then loaded on acrylamide gel and underwent standard western blotting procedures to analyze GluA2/3, GluA1, tubulin, and transferrin receptor expression.

### Western Blot and Antibodies

For the analysis of synaptic marker expression, hippocampi from WT and Myo9a^+/-^ mice were dissected and homogenized in RIPA buffer (20 mM Tris, 150 mM NaCl, 1 mM EDTA, 1% NP40, 1% Triton X-100, protease inhibitors, pH 7.5). Ten micrograms of protein from each sample were loaded onto an acrylamide gel for western blotting_._

Primary antibodies directed against the following proteins were used: Myo9a (Tü 76, WB 1:1000; Tü 78, IP 10 μg/ml; Chieregatti et al., 1998; Hanley et al., 2010), Myo5a (AbCAM, 1:1000), GluA1 (Chemicon, 1:1000), GluA2 (Chemicon, 1:1000), GluA2/3 (1:2000, gift from C. Gotti), GluK2 (Prestige, 1:1000), Homer1 (Santa Cruz, 1:500), PSD95 (Neuromab, 1:20000), Synaptophysin1 (SySy, 1:5000), GAPDH (Santa Cruz, 1:1000), *N*-cadherin (NCAD) (AbCAM, 1:1000), PICK1 (Neuromab, 1:1000), GRIP1 (BD Transduction Laboratories, 1:2000), Tubulin (Sigma, 1:50000), Transferrin receptor (Invitrogen, 1:1000), VGAT (SySy, 1:1000), and VGLUT (SySy, 1:2000). The secondary antibodies, horseradish peroxidase- (Sigma) or IR Dyes- (LI-COR) conjugated antibodies, were used for western blots. Samples were separated using SDS-PAGE, and western blots then visualized with the Pierce ECL-Detection Kit or an Odyssey Infrared Imager (LI-COR).

### Golgi Staining

Mice were anesthetized by intraperitoneal injection of 10 mg/ml avertine and transcardially perfused with 100 ml of saline solution (0.9% NaCl). After perfusion, dissected brains were plunged in Golgi-Cox Solution (potassium dichromate 1%, mercuric chloride 1%, and potassium chromate 0.8%) for 1 week in the dark at room temperature. Subsequently, brains were washed in water several times to remove the Golgi-Cox Solution and incubated in a 30% sucrose solution for at least 2 days in the dark at 4°C. Coronal sections (100 μm thickness) were obtained with a vibratome (Leica VT1000S) and collected on gelatinized slides. After 24 h, brain slices were stained in the dark. Briefly, slices were washed twice with water and embedded for 30 min in ammonium hydroxide. Rinsed slides were fixed for 30 min in Kodak fix solution. Subsequently slices were incubated with increasing concentrations of ethanol (50, 70, 95, and 100%) for 13 min, then a solution composed by 1/3 chloroform, 1/3 xylene, and 1/3 ethanol for 15 min, and xylene for 15 min. Finally slides were mounted with a coverslip using Permount mounting medium (Fisher Scientific). Acquisition of the stained neurons from the CA1 region of hippocampus was performed using a Spinning Disk Confocal Microscope. Stacks were collected every 0.5 μm using 20× or 63× lens. Analysis of the dendritic spine density was performed on 1500 μm of dendrites (63× images) using NeuroStudio software.

### Electron Microscopy

Mice were anesthetized by an intraperitoneal injection of 10 mg/ml avertine and transcardially perfused with 2.5% glutaraldehyde and 2% paraformaldehyde in 0.15 M sodium cacodylate buffer (pH 7.4). Dissected brains were post-fixed for an additional 24 h at 4°C. Coronal sections (100 μm thickness) were obtained with a vibratome (Leica VT1000S), and hippocampi were manually dissected. After washing, samples were post-fixed with 2% osmium tetroxide, rinsed, stained with 1% uranyl acetate in water for 45 min, dehydrated and embedded in epoxy resin (Electron Microscopy Science, Hatfield, PA, USA) that was baked for 48 h at 60°C. Thin sections were obtained with an ultramicrotome (Leica Microsystems, Austria), stained with a saturated solution of uranyl acetate in ethanol 20% and observed under a Philips CM10 transmission electron microscope (TEM) (FEI, Eindhoven, Netherland). For quantitative analyses, images were acquired at a final magnification of 25–34000× using a Morada CCD camera (Olympus, Munster, Germany).

#### Quantitative Analysis of TEM Images

Excitatory synapses in the apical dendrite layer of the hippocampal CA1 region were identified and selected for analyses based on the presence, in their postsynaptic terminal, of the electron-dense PSD. We calculated the synaptic vesicle (SV) density and density of docked SV, the area of the presynaptic button, the density of excitatory synapses and the length and thickness of the PSD. The average thickness of the PSD was calculated as described in Dosemeci et al. (2001). The estimation of the numerical density of excitatory synapses per μm^3^ of hippocampus was calculated using a size-frequency stereological method (DeFelipe et al., 1999). A total of 18 images (25000×, 20.28 μm^2^ each for a total of 365 μm^2^) were analyzed for each genotype. Analyzed images were not from serial or nearby sections and included exclusively apical CA1 dendrites, while images with cell bodies, axons or non-apical dendrites were discarded. Images were analyzed with ImageJ version 1.47 m (NIH Image).

### Electrophysiology

Hippocampal slices were prepared from WT and Myo9a^+/-^ male mice. Mice were anesthetized in a chamber saturated with chloroform and then decapitated. The brain was rapidly removed and placed in an ice-cold solution at pH 7.3, equilibrated with 95% O_2_ and 5% CO_2_ [for patch-clamp recordings: 220 mM sucrose, 2 mM KCl, 1.3 mM NaH_2_PO_4_, 12 mM MgSO_4_, 0.2 mM CaCl_2_, 10 mM glucose, 2.6 mM NaHCO_3_, 3 mM kynurenic acid; for field excitatory post synaptic potentials (fEPSPs) recordings: artificial cerebrospinal fluid (aCSF) containing 125 mM NaCl, 2.5 mM KCl, 1.25 mM NaH_2_PO_4_, 1 mM MgCl_2_, 2 mM CaCl_2_, 25 mM glucose, and 26 mM NaHCO_3_].

The cerebellum and prefrontal cortex were removed before cutting and coronal brain slices (thickness: 300 μm for patch-clamp recordings and 400 μm for fEPSPs) containing the hippocampal formation were made using a vibratome (Leica VT1000S). Immediately after the cutting procedure, slices were incubated at 37°C for 40 min and then at room temperature for 1 h in standard aCSF solution before recordings.

Slices were transferred to a recording chamber perfused with aCSF at a rate of ∼2 ml/minute at room temperature. Whole-cell patch-clamp and fEPSPs recordings were performed using a Multiclamp 700B amplifier (Axon CNS molecular devices, USA) and an infrared-differential interference contrast microscope. Patch microelectrodes (borosilicate capillaries with a filament and an outer diameter of 1.5 μm, Sutter Instruments) were prepared with a four-step horizontal puller (P-1000, Sutter Instruments) and had a resistance of 3–5 MΩ.

Miniature excitatory postsynaptic currents (mEPSCs) were recorded from pyramidal cells of the hippocampal CA1 region at a holding potential of -65 mV with an internal solution containing 126 mM *K*-gluconate, 4 mM NaCl, 1 mM EGTA, 1 mM MgSO_4_, 0.5 mM CaCl_2_, 3 mM ATP (magnesium salt), 0.1 mM GTP (sodium salt), 10 mM glucose and 10 mM HEPES-KOH, pH 7.28, osmolarity adjusted to 280 mOsm.

For paired pulse experiments, two excitatory postsynaptic currents (EPSCs) were evoked by paired stimulations of Schaffer’s collaterals using a glass pipette filled with aCSF and placed at 100/200 μm from the recorded CA1 pyramidal neuron (interpulse interval 40 ms). The paired pulse ratio (PPR) was computed from the average of 20 consecutive episodes of stimulation and was calculated dividing the second response (P2) by the first one (P1). Access resistance was between 10 and 20 MΩ; if it changed by >20% during the recording, the recording was discarded.

Evoked EPSCs were recorded in the presence of bicuculline (20 μM); mEPSCs were recorded in the presence of bicuculline plus lidocaine (500 μM) in the aCSF.

For fEPSPs recordings, Schaffer-collateral fiber bundles were stimulated using a glass pipette filled with aCSF and placed at 200/300 μm from the recording electrode in the stratum radiatum to evoke a half maximal response. After 10 min of stable baseline (fEPSPs evoked every 20 s), LTP was induced stimulating Schaffer-collaterals pathway with one train of 100 stimuli at 250 Hz according to Talani et al. (2011). Currents and potentials were filtered at 2 kHz through the amplifier and digitized at 20 kHz using Clampex 10.1 software. The analysis was performed oﬄine with Clampfit 10.1 software.

### Behavioral Tests

Male mice of 2–3 months of age were housed individually in polycarbonate cages with food and water freely available through wire lids. Cob bedding was changed weekly, and the vivarium was 21°C with a 12 h light cycle (lights on at 08:00 a.m.). All of the behavioral tests were carried out between 9:00 a.m. and 1:00 p.m.

#### General Health Assessment

To ascertain that mice were healthy, body weight, and food intake were monitored weekly during behavioral experiments.

#### Spontaneous Motor Activity

Spontaneous motor activity was evaluated in an automated activity cage (43 cm × 43 cm × 32 cm) (Ugo Basile, Varese, Italy) placed in a sound-attenuating room as previously described (Ferri et al., 2004). Cumulative horizontal and vertical beam breaks were counted for 30 min.

#### Visual Cliff

Visual acuity was tested in the visual cliff paradigm according to Van’t Veer et al. (2013). The apparatus consisted of a platform with a checkered pattern positioned 1 m above the ground. A clear piece of Plexiglas was placed on the platform and extended 0.5 m beyond the platform edge. A checkered pattern was also placed on the floor below the extending Plexiglas, creating the illusion of a sudden drop-off. At the interface between the shallow side and the deep side, there was a ridge of aluminum (1 cm). Each mouse was placed on the aluminum ridge and was allowed to step off to either side. Choices were manually recorded as safe if the mouse stepped towards the platform side (good visual acuity) and as unsafe if the mouse chose the overhang side (poor visual acuity). Each mouse was tested in 10 trials.

#### Swimming Tank Test

To monitor swimming movements, mice were trained to swim from one end of a water-filled glass tank to a visible escape platform at the opposite end (Perry et al., 1995). The glass tank was 100 cm long and 6 cm wide and was filled to a depth of 20 cm with water at a temperature of 23°C. The visible escape platform was made from black perspex (6 cm square and 20.5 cm high), with the top surface 0.5 cm above the water level. A vertical black line on the side of the glass marked a horizontal distance 60 cm from the platform; this served as the start line for recording swimming performance. During the training period, each mouse was placed in the water at one end of the tank and within a couple of trials, learned to swim straight to the visible escape platform at the opposite end. After training, mice were given two trials per day for three consecutive days, by which time they reached stable baseline performance levels. Mice were then given two trial tests during which mice were videotaped from both sides, and the number of forelimb kicks, the number of hindlimb kicks, the latency to swim the 60 cm distance and the swimming speed were recorded using a SpeedClock iPhone application. Analysis was based on the mean scores of the two trials for each measure.

#### Spatial Object Recognition

Spatial object recognition occurred in an open field white arena (58 cm × 50 cm × 43 cm). Two visual cues were placed on two adjacent walls of the open field arena according to Kenney et al. (2011). Specifically, a black and white striped pattern was affixed to the center of the northern wall, and a black and gray checkered pattern was placed at the center of the western wall. The animals were first habituated to the test apparatus for 10 min for one day. Then, they were subjected to a familiarization trial (T1) and a spatial object recognition trial (T2) after 120 min. During T1, mice were placed into the open field arena for 10 min and allowed to explore two different objects placed in the NE and NW corners. Objects were placed approximately 5 cm from the walls of the open field arena. Then the mice were returned to the home cage. After 2 h, mice were re-exposed for 10 min to the open field arena where the object that was in the NW corner was moved to the SW corner. Objects were counterbalanced across locations and conditions. The open field arena and all of the objects were thoroughly wiped with 70% ethanol before and after all of the behavioral procedures. Care was taken to minimize the difference between the objects in order to prevent a preference for one of them. An experimenter blind to the mouse genotype manually recorded the exploration time. Mice that did not explore any of the objects for at least 30 s during T1 were excluded from the analysis. The time spent by mice exploring each object during T1 and T2 was measured, and the discrimination index [(time spent exploring novel object - time exploring familiar object)/(time spent exploring novel object + time exploring familiar object)] was calculated as described in Pitsikas et al. (2001).

#### *T*-Maze

Mice were deprived of food until they had reached 85–90% of their free-feeding body weight. Mice were habituated to a black wooden *T*-maze and trained to obtain food across the maze for 5 days (Sala et al., 2011). The *T*-maze had a 41 cm stem section and a 91 cm arms section; each section was 11 cm wide and had walls 19 cm high. In the acquisition phase, one arm was designated as the reinforcer (Coco pops; Kellogg’s) for each of ten daily trials. Each mouse was placed at the start of the maze and was given a free choice to enter either arm. The number of days to reach the criterion (80% of correct choices for 3 days) was recorded. Each mouse that met the criterion for acquisition was then tested using a reversal procedure in which the reinforcer arm was changed.

#### Morris Water Maze

To evaluate the integrity of hippocampus-dependent memory formation in WT and Myo9a^+/-^ mice, the Morris water maze paradigm according to Morris et al. (1982) was used. The water maze was a circular tank (diameter 1.5 m) filled with water (25 ± 0.5°C). A platform (13.5 cm × 23 cm) was submerged below the water’s surface in the center of the target zone. Floating polystyrene particles were placed on the surface of the water to hide the platform from sight. Extra maze cues (simple geometrical shapes) and visual references (chair, table) were present around the room to provide spatially oriented cues. Intra-maze cues along the edge of the pool were also present. Four points around the circumference of the maze were arbitrarily designated as N, S, E, and W, which served as a reference for experimenters when releasing the mice into the pool. Within each acquisition session (i.e., four trials in a day), mice were randomly released from each of the four points. After one day of habituation in the maze for 60 s without the platform, acquisition training was performed. Acquisition consisted of four trials per day for four consecutive days and an inter-trial interval of 1 h. Once the mouse located the hidden platform, the mouse remained on the platform for 30 s before removal from the tank. If a mouse failed to locate the platform within 120 s, it was guided to the platform and remained there for 30 s before removal from the pool. On the sixth day, the platform was removed (probe test), and the animals were allowed to remain in the pool for 120 s. Trials were recorded using a digital video camcorder (Canon MV900) fixed to the ceiling. An experimenter, blind to the genotype, manually recorded the escape latency, the time spent in the quadrants of the maze and the number of target quadrant crossings.

### Statistical Analysis

Data were expressed as the mean ± SEM. An *F*-test was used for variance comparison and was followed by a non-parametric Welch’s *t*-test (unequal variances) or a two-tailed unpaired Student’s *t*-test (equal variances). Multiple group comparisons were performed either by one- or two-way analysis of variance (ANOVA) followed by Tukey’s or Bonferroni’s *post hoc* tests. The results were considered significant if *p* < 0.05. All of the statistical analyses were performed using Prism 6 software (GraphPad, San Diego, CA, USA).

#### Number of Replicates and Number of Animals Used

Each *in vitro* experiment was repeated at least three times. Primary neurons from three independent preparations were used. The following numbers (*N*) of WT and Myo9a^+/-^ mice were analyzed: Golgi staining (**Figure 2A**) *N* = 3, 10 neurons; EM (**Figures 2B,C**) *N* = 3, 40 presynaptic terminals, 80 PSDs; biochemical assays (**Figures 3A,B**) *N* = 4; BS^3^ crosslinking experiments (**Figures 3C,D**) *N* = 3; mEPSCs and paired pulse recordings (**Figures 4A,B**) *N* = 8; LTP (**Figure 4C**) *N* = 3; and behavioral tests (**Figures 5** and **6**) *N* = 5–7.

## Results

### Myo9a Localizes to the PSD and Directly Interacts with the AMPAR Subunit GluA2

**FIGURE 1:**
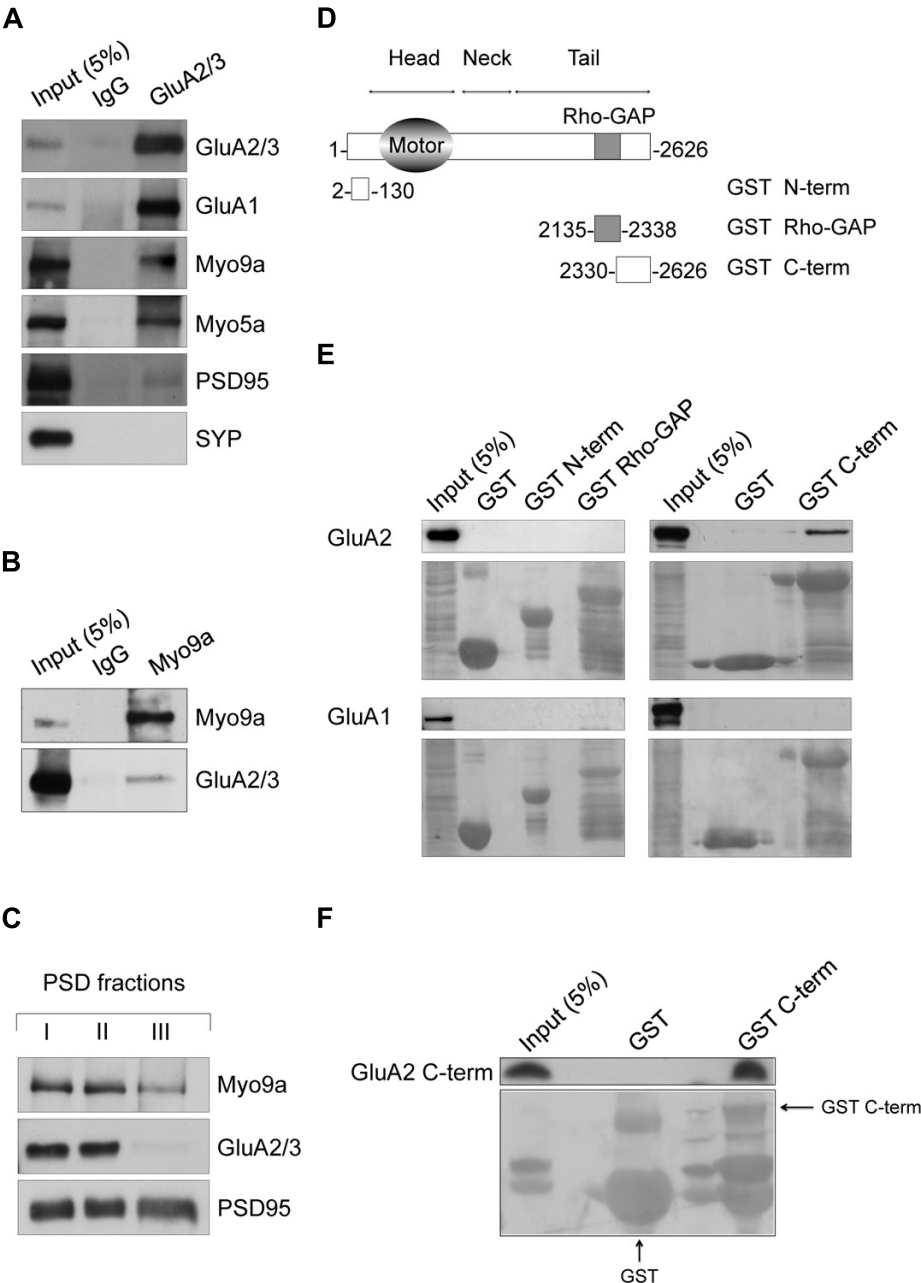
**Myosin IXa (Myo9a) localizes at excitatory synapses and binds the alpha-amino-3-hydroxy-5-methyl-4-isoxazole propionic acid receptor (AMPAR) GluA2 subunit. (A)** Western blots of co-immunopurified proteins from DIV18 hippocampal neurons using anti-GluA2/3 antibodies or non-immune IgGs as a control. GluA1, Myo9a, Myo5a, and reduced amounts of PSD95 were co-purified with GluA2/3, but SYP was not. **(B)** Co-immunoprecipitation of GluA2/3 with Myo9a from mouse hippocampal homogenates. **(C)** Biochemical fractionation of Myo9a in rat brain showing the presence of Myo9a in postsynaptic density (PSD) fractions that were extracted with Triton X-100 once (PSD I) and twice (PSD II). Myo9a was reduced following extraction with Triton X-100 followed by sarkosyl (PSD III). **(D)** Schematic structure of Myo9a and its domains fused to GST. **(E)** Western blots of pull-down assays from HEK293 cells expressing either GluA2 (top) or GluA1 (bottom) using GST N-term, GST Rho-GAP and GST C-term or GST as a control. Myo9a C-terminal region (GST C-term) binds GluA2 but not GluA1. Note that a blank line was left between each sample line to avoid overflow artifacts. A small amount of overflow into the adjacent wells appears as additional narrow lines. **(F)** *In vitro* pull-down assays of purified GluA2 C-terminus (GluA2 C-term) using Myo9a GST C-term, which indicates that Myo9a and GluA2 C-termini directly interact. GST fusion proteins were visualized using Ponceau Red staining (underneath each immunoblot). GST fusion proteins are indicated using arrows. Small amount of overflow into the adjacent wells appear as additional narrow lines.

Our study is based on the finding that Myo9a and AMPAR are associated in the same molecular complex in hippocampal neurons. We used anti-GluA2/3 antibodies to immunopurify AMPARs and their interacting proteins from primary cultures of rat hippocampal neurons (**Figure 1A**). As most AMPARs in hippocampal pyramidal neurons are composed of either GluA2-GluA1 or GluA2-GluA3 (Wenthold et al., 1996), anti-GluA2/3 antibodies allow for the purification of the large majority of AMPARs. Interestingly, we detected Myo9a among the co-purified proteins. As expected, the GluA1 AMPAR subunit and Myosin Va (Myo5a), which is a GluA1 C-terminus binding partner (Correia et al., 2008), were also identified. PSD95, which does not directly interact with AMPAR, was poorly co-purified, and the presynaptic protein synaptophysin (SYP) was not observed (**Figure 1A**), thus demonstrating the specificity of the purified proteins. In addition to these results, the precipitation of Myo9a with a specific antibody from mouse hippocampal homogenates allows for the co-immunoprecipitation of GluA2/3 (**Figure 1B**). In neurons, AMPARs are enriched at the PSD of dendritic spines where they mediate most of the fast excitatory transmission (Bredt and Nicoll, 2003). The association of Myo9a with AMPARs in biochemical assays suggests that Myo9a might be a component of the PSD. To validate this hypothesis, we prepared PSD fractions from the rat brain and compared the distribution of Myo9a with that of the PSD proteins PSD95 and AMPAR (**Figure 1C**). PSD95, which is the typical marker of the PSD, is fully resistant to both Triton X-100 (PSD I and II) and sarkosyl (PSD III) extraction. AMPAR, which is less tightly associated with the PSD, is present in PSD fractions I and II but is largely extracted by sarkosyl treatment (note the absence of the GluA2/3 signal in the PSD III fraction). Myo9a is detectable in all of the PSD fractions including PSD III, indicating a strong association with the PSD, and it is enriched in the GluA2/3 positive fractions (PSD I and II), consistently with a Myo9a- AMPAR association (**Figure 1C**).

Next, to better characterize the AMPAR-Myo9a association, we mapped their interacting regions (**Figures 1D–F**). We tested three Myo9a regions fused with the GST protein for interactions with either GluA1 or GluA2 in transfected HEK293 cells, namely, the N-terminal extension specific of class IX myosins (GST N-term), the Rho-GAP domain (GST Rho-GAP) and the C-terminus (GST C-term) (**Figures 1D,E**). Only the Myo9a C-terminus was able to precipitate GluA2 (**Figure 1E**, top). Interestingly GluA1 was not precipitated by any of the GST fusion proteins, indicating that Myo9a specifically binds to the AMPAR subunit GluA2 (**Figure 1E**, bottom).

AMPAR subunits are characterized by different intracellular C-termini that bind to several proteins that are required for the trafficking and synaptic expression of the receptor (Shi et al., 2001). We therefore investigated whether Myo9a binds the GluA2 C-terminal tail and whether this interaction is direct (**Figure 1F**). Toward this end, we evaluated the Myo9a GST C-terminus in a pull-down assay with the purified GluA2 C-terminus (GluA2 C-term). The Myo9a C-terminus was able to precipitate the GluA2 C-term, which demonstrates that the C-termini of Myo9a and GluA2 interact directly (**Figure 1F**).

Altogether, these results indicate that Myo9a is a novel binding partner of the AMPAR subunit GluA2, and it is expressed at the PSD.

### Myo9a^+/-^ Mice Display a Thicker PSD and Increased PSD95 and Surface AMPAR Levels

Because Myo9a is present in PSDs and interacts with GluA2, we hypothesized a role of Myo9a at synapses in regulating glutamatergic signaling and decided to investigate the effect of Myo9a deficiency on hippocampal synapses *in vivo*.

Myo9a homozygous knockout mice (Myo9a^-/-^) are characterized by high lethality, develop severe hydrocephalus within the first 2 weeks of life and display major motor defects (Abouhamed et al., 2009). In contrast, heterozygous mice (Myo9a^+/-^) grow normally, their brain architecture is preserved and they do not show locomotor defects (Abouhamed et al., 2009 and data not shown). For this reason, we decided to use Myo9a^+/-^ in our study. Indeed Myo9a^+/-^ mice represent a model that is suitable for a comprehensive set of structural, electrophysiological, and behavioral analyses.

**FIGURE 2:**
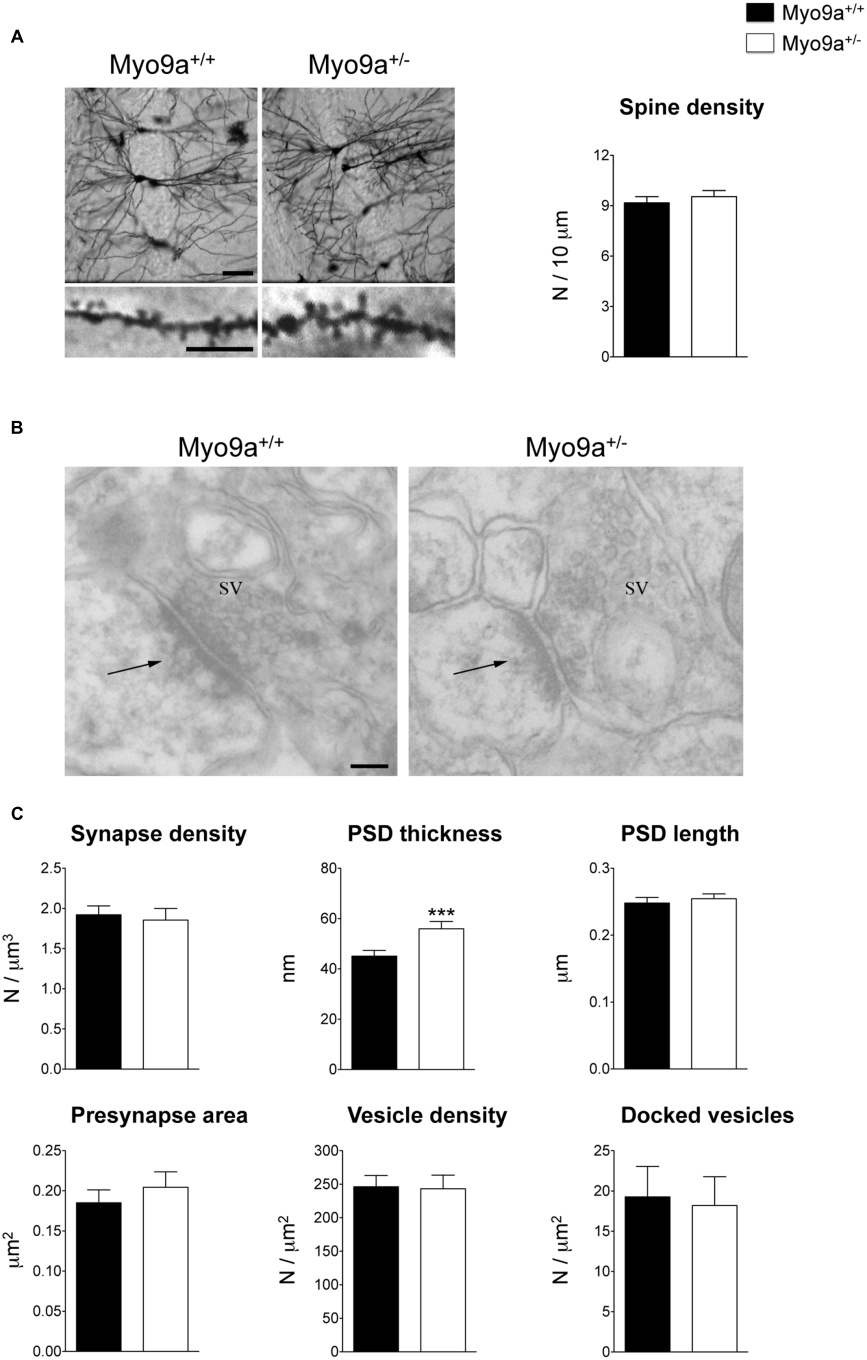
**Myo9a^+/-^ mice displayed a thicker PSD compared to WT mice. (A)** Representative images of Golgi staining of hippocampal CA1 pyramidal neurons (scale bar 50 μm) and apical dendrites segments (scale bar 5 μm) from Myo9a^+/+^ (WT) and Myo9a^+/-^ mice (left). Dendritic spine density measurement is shown (right). **(B)** Representative electron micrographs of asymmetrical synapses of CA1 *stratum radiatum* from WT and Myo9a^+/-^ mice hippocampi. Arrows indicate the PSDs (scale bar 100 nm). **(C)** Quantification of EM images and relative histograms. Excitatory synapse densities did not differ between genotypes. PSD thickness is significantly increased in Myo9a^+/-^ mice, while PSD length, the area of the presynaptic button, the density of total and docked synaptic vesicles (SV) did not vary between genotypes.

We started by performing morphological analyses of the excitatory synapses of the hippocampus focusing on the best-characterized CA1 area (**Figure 2**). First, we evaluated the number of dendritic spines on Golgi stained dendrites from Myo9a^+/-^ and WT mice. The two groups of mice had a similar number of spines (WT 9.171 ± 0.368 vs. Myo9a^+/-^ 9.538 ± 0.362 spines/10 μm; **Figure 2A**). Notably, the stereological analysis of EM images reconfirmed these data (WT 1.922 ± 0.111 vs. Myo9a^+/-^ 1.855 ± 0.145 synapses/μm^3^; **Figures 2B,C**). Next, we assessed the morphology of pre- and post-synaptic compartments. The area of presynaptic terminals and the density of both total and docked SVs were the same in both genotypes (presynaptic button: WT 0.185 ± 0.016 vs. Myo9a^+/-^ 0.204 ± 0.019 μm^2^; total SV density: WT 246.3 ± 16.89 vs. Myo9a^+/-^ 243.4 ± 20.14 vesicles/μm^2^; docked SV density: WT 19.27 ± 3.79 vs. Myo9a^+/-^ 18.18 ± 3.59 vesicles/μm^2^). Interestingly, Myo9a^+/-^ mice displayed a thicker PSD than WT mice (WT 44.65 ± 1.06 vs. Myo9a^+/-^ 59.88 ± 1.88 nm, ^∗∗∗^*p* < 0.001), while PSD length did not vary between genotypes (WT 0.248 ± 0.008 vs. Myo9a^+/-^ 0.254 ± 0.007 μm; **Figures 2B,C**).

**FIGURE 3:**
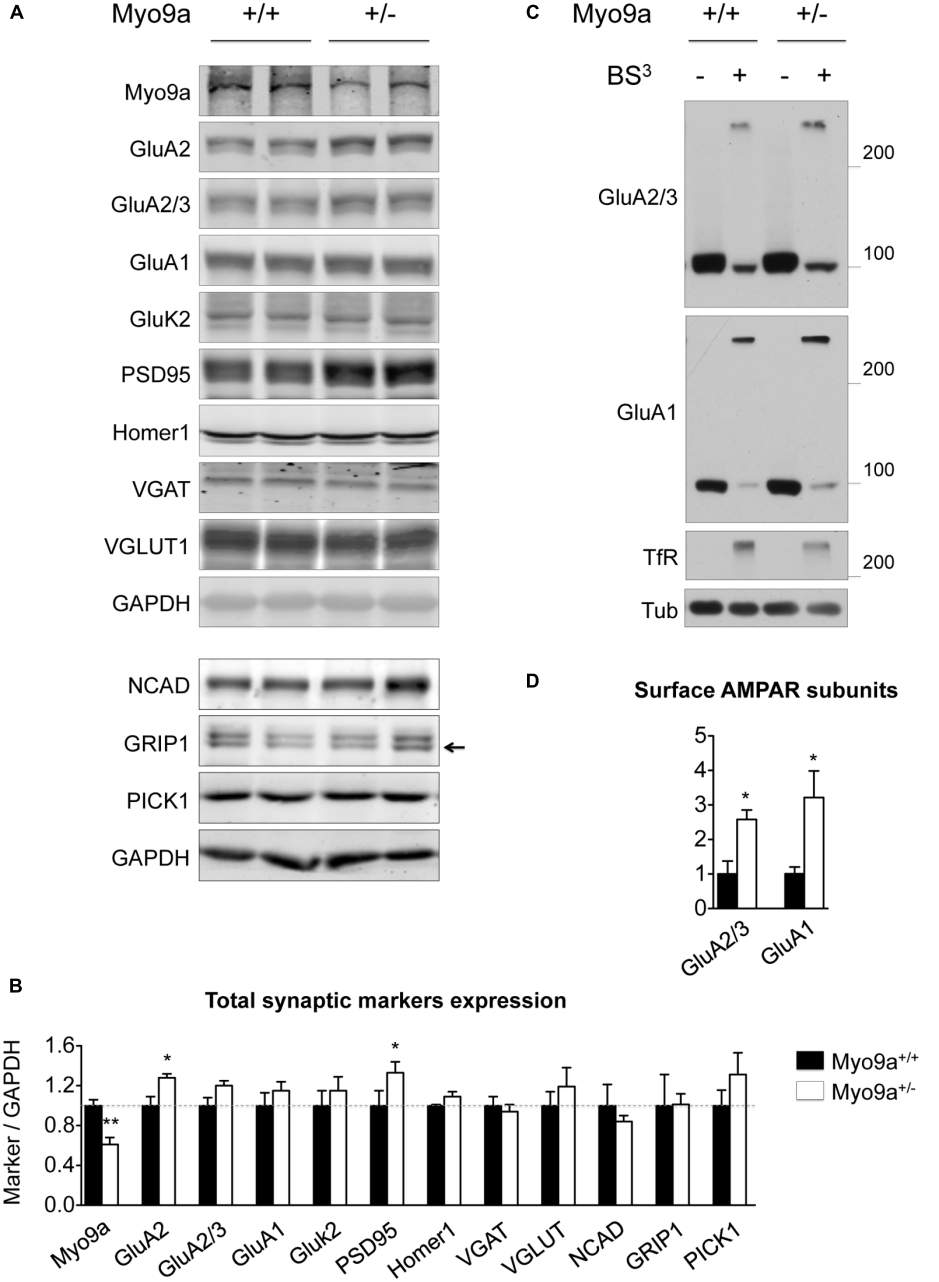
**Myo9a^+/-^ mice displayed increased GluA2 and PSD95 expression levels and increased AMPAR surface content. (A,B)** Immunoblots from hippocampal homogenates from WT and Myo9a^+/-^ mice using LI-COR technology **(A)** and histograms showing the quantification of synaptic markers **(B)**. Myo9a^+/-^ mice, retaining approximately half of the Myo9a protein level, expressed higher levels of GluA2 and PSD95 compared with WT, but other postsynaptic and presynaptic markers were unvaried. **(C)** Immunoblots from BS^3^ crosslinking on mouse brain slices to detect surface AMPAR pools. Both high (surface-expressed) and predicted (intracellular) molecular weight bands were detected in tissue that was treated with the crosslinker (BS^3^+), whereas surface proteins from untreated tissues (BS^3^–) and intracellular proteins (tubulin) yielded single bands. **(D)** Quantification of surface AMPAR subunits normalized to surface transferrin receptor (TfR) is shown. In Myo9a^+/-^ mice, surface GluA2/3 and GluA1 levels were higher than those of WT mice.

Because PSD size correlates with PSD protein content (Takumi et al., 1999), we evaluated whether the thicker PSDs of Myo9a^+/-^ mice were associated with a higher expression of synaptic markers in the hippocampus (**Figures 3A,B**). As expected, Myo9a^+/-^ mice were characterized by a strong reduction of Myo9a expression (WT 1 ± 0.06 vs. Myo9a^+/-^ 0.61 ± 0.07, ^∗∗^*p* < 0.01). Notably, reduced expression of Myo9a was associated with a significant increase in PSD95, which is the major scaffolding protein of the PSD (WT 1 ± 0.15 vs. Myo9a^+/-^ 1.33 ± 0.11, ^∗^*p* = 0.01) and of the Myo9a binding partner GluA2 (WT 1 ± 0.09 vs. Myo9a^+/-^ 1.28 ± 0.04, ^∗^*p* = 0.03). In contrast, AMPAR subunits GluA2/3 and GluA1, the GluA2 interacting proteins NCAD, GRIP1, and PICK1, the kainate receptor subunit GluK2, the scaffolding protein Homer1, and the presynaptic vesicular markers VGAT and VGLUT were unaltered (GluA2/3: 1 ± 0.08 vs. Myo9a^+/-^ 1.2 ± 0.05; GluA1: 1 ± 0.13 vs. Myo9a^+/-^ 1.15 ± 0.09; GluK2: 1 ± 0.15 vs. Myo9a^+/-^ 1.15 ± 0.14; Homer1: 1 ± 0.01 vs. Myo9a^+/-^ 1.09 ± 0.05; VGAT: 1 ± 0.09 vs. Myo9a^+/-^ 0.94 ± 0.07; VGLUT1: 1 ± 0.14 vs. Myo9a^+/-^ 1.19 ± 0.19; NCAD: 1 ± 0.21 vs. Myo9a^+/-^ 0.84 ± 0.05; GRIP1: 1 ± 0.31 vs. Myo9a^+/-^ 1.01 ± 0.10; PICK1: 1 ± 0.15 vs. Myo9a^+/-^ 1.31 ± 0.21) (**Figures 3A,B**).

Next, we determined whether an AMPAR increase occurred at the neuronal surface. BS^3^ crosslinking experiments allowed us to quantify the surface expression of membrane proteins and showed that Myo9a^+/-^ mice displayed more GluA2/3- and GluA1-containing AMPARs on the plasma membrane than WT mice (GluA2/3: 1 ± 0.37 vs. Myo9a^+/-^ 2.57 ± 0.27, ^∗^*p* = 0.02; GluA1: 1 ± 0.08 vs. Myo9a^+/-^ 3.21 ± 0.76, ^∗^*p* = 0.04) (**Figures 3C,D**).

Overall, these data indicate that Myo9a haploinsufficiency did not affect the density of synaptic contacts or the structure of presynaptic terminals. In contrast, reduced levels of Myo9a specifically altered the morphology of the PSD by increasing PSD thickness. Concomitantly, the expression of the scaffolding protein PSD95 and the GluA2 AMPAR subunit was increased in addition to the amount of AMPARs on the plasma membrane.

### Myo9a Haploinsufficiency Impairs Hippocampal Synaptic Transmission and LTP

**FIGURE 4:**
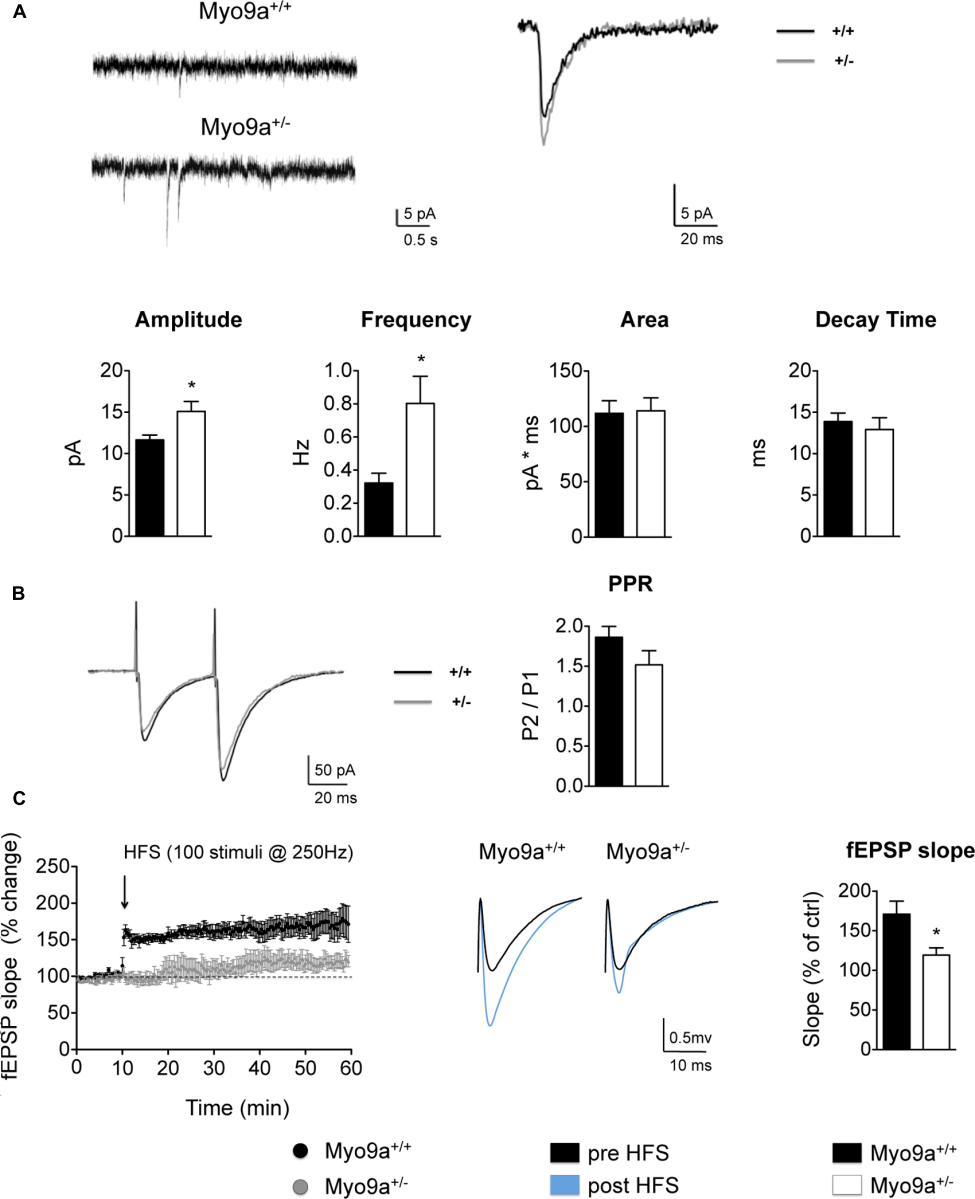
**Myo9a haploinsufficiency affected basal AMPAR transmission and impaired LTP. (A)** Representative mEPSC recordings (top, left) and average traces (2 min) (top, right) from WT and Myo9a^+/-^ hippocampal slices in the presence of 500 μM of lidocaine and 20 μM of bicuculline. Heterozygous mice showed a significant increase in mEPSCs amplitude and frequency, whereas currents area and decay time were unvaried (bottom). **(B)** Representative current traces that were evoked by paired stimuli (left) and PPR quantification (right) indicated no differences between genotypes. **(C)** Quantification of field excitatory post synaptic potentials (fEPSP) slopes before and after high frequency stimulation (HFS, left). Representative traces from WT and Myo9a^+/-^ mice are shown (middle). The histograms show the fEPSP slope of the last 10 min of LTP induction (right), which revealed that LTP induction is reduced in Myo9a^+/-^ mice.

Increasing AMPAR surface levels means enhancing the receptive potential of a synapse, as more receptors will be available to respond to the glutamate released by the presynapse. Therefore, we investigated the spontaneous glutamatergic transmission in CA1 pyramidal neurons of hippocampal slices by measuring mEPSCs (**Figure 4A**). As expected, Myo9a^+/-^ mice displayed increased mEPSC amplitude compared with WT mice, indicating that more AMPARs were added to individual synapses (WT 11.6 ± 0.6 vs. Myo9a^+/-^ 15.1 ± 1.2 pA, ^∗^*p* = 0.03). In contrast, area and decay time of mEPSCs were unvaried between genotypes (area: WT 111.8 ± 11.4 vs. Myo9a^+/-^ 114.1 ± 11.7 pA ^∗^ ms; decay time: WT 13.9 ± 1.0 vs. Myo9a^+/-^ 12.9 ± 1.4 ms), suggesting that there were no changes in AMPAR composition at the surface, which is in agreement with an increase of both GluA1 and GluA2 containing AMPARs on the plasma membrane.

Notably, Myo9a^+/-^ mice also displayed increased mEPSCs frequency (WT 0.32 ± 0.06 vs. Myo9a^+/-^ 0.80 ± 0.16 Hz, ^∗^*p* = 0.04, **Figure 4A**), which could have been due to an increase of presynaptic neurotransmitter quanta release or was a consequence of postsynaptic AMPARs enrichment. To discriminate between these two possibilities, we measured paired pulse facilitation in Myo9a^+/-^ and WT mice (**Figure 4B**). In these measurements, we observed no differences between genotypes (WT 1.86 ± 0.13 vs. Myo9a^+/-^ 1.52 ± 0.18) and excluded a presynaptic effect.

Our data suggested that the mEPCS frequency increase originated from postsynaptic modifications. In particular, the increase in frequency might by due to the presence of more dendritic spines in Myo9a^+/-^ mice or can be secondary to the increased amplitude of the mEPSCs. Because the excitatory synapse density, as evaluated by Golgi staining and EM, was comparable between Myo9a^+/-^ and WT mice, we propended for the second hypothesis. We postulated that more synapses in heterozygous mice contained a sufficient amount of surface AMPARs to render their currents detectable. Indeed, the increased amplitude of mEPSCs allows for the quantification of some mEPSCs that normally fall below the detection threshold, resulting in an increased frequency.

Defects in the mechanisms regulating the expression and distribution of synaptic proteins are expected to compromise not only the basal properties of a synapse but also its plastic remodeling in response to external stimuli. Indeed, synaptic strengthening or weakening relies on a tight regulation of the amount of synaptic proteins, particularly the surface pool of AMPARs (Huganir and Nicoll, 2013). This prompted us to investigate whether LTP was affected by Myo9a haploinsufficiency. Notably, LTP induction at the CA3–CA1 synapse was impaired in Myo9a^+/-^ mice compared to WT littermates (fEPSP slope, last 10 min: WT 171 ± 16.49 vs. Myo9a^+/-^ 119.2 ± 9.282%, ^∗^*p* = 0.02) (**Figure 4C**).

Together these results implicate Myo9a involvement in both basal synaptic transmission and plasticity of the excitatory synapses of the hippocampus.

### Myo9a Haploinsufficiency Impairs Learning and Memory

**FIGURE 5:**
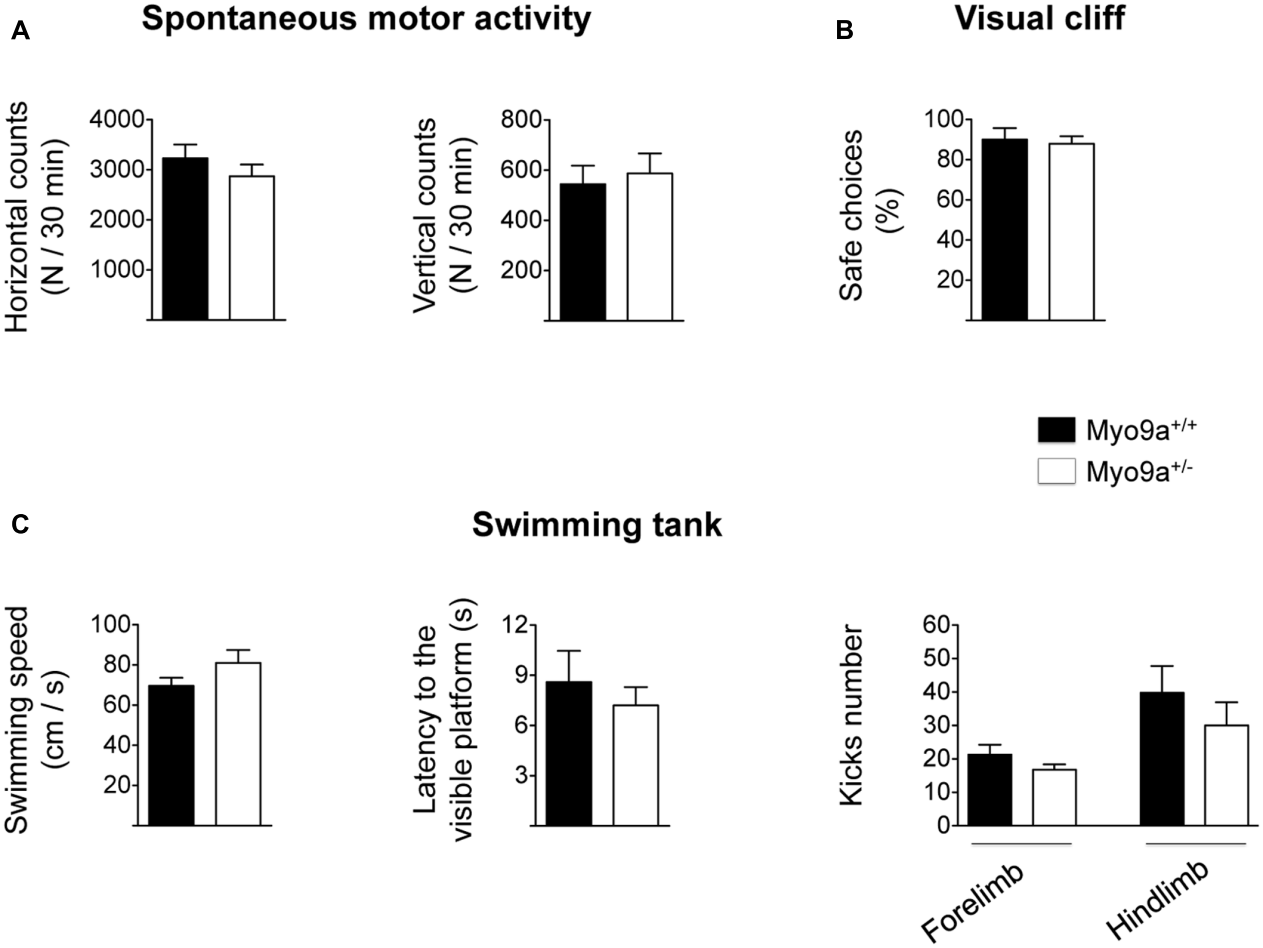
**Myo9a haploinsufficiency does not affect spontaneous motor activity, visual acuity, and swimming capability. (A)** WT and Myo9a^+/-^ mice show comparable spontaneous motor activity in terms of the number of horizontal (left) and vertical (right) movements that were evaluated in an automated activity cage for 30 min. **(B)** In a visual cliff paradigm WT and Myo9a^+/-^ mice made the same percentage of safe choices, indicating good visual acuity in both animal groups. **(C)** Swimming activity of WT and Myo9a^+/-^ was evaluated by measuring swimming speed (left), latency to the visible platform (middle) and the number of forelimb and hindlimb kicks (right). No differences were detected between genotypes.

As LTP has been proposed as the biological substrate for learning and memory (Teyler and Discenna, 1984), we tested Myo9a^+/-^ mice in hippocampal-dependent cognitive paradigms. We first found that, in contrast to Myo9a^-/-^ littermates, Myo9a^+/-^ mice displayed normal spontaneous activity, as measured in an activity cage (mean number of horizontal counts in 30 min: WT 3230 ± 274 vs. Myo9a^+/-^ 2870 ± 234; mean number of vertical counts in 30 min: WT 544 ± 73 vs. Myo9a^+/-^ 587 ± 79) (**Figure 5A**). Furthermore, Myo9a^+/-^ mice showed normal visual acuity, which was evaluated by the visual cliff paradigm (safe choices: WT 90 ± 5.7 vs. Myo9a^+/-^ 88 ± 3.7%) (**Figure 5B**), and normal swimming behavior (swimming speed: WT 69.6 ± 4.06 vs. Myo9a^+/-^ 81 ± 6.47 cm/s; latency to the visible platform: WT 8.6 ± 1.85 vs. Myo9a^+/-^ 7.2 ± 1.1 s; forelimb kicks number: WT 21.25 ± 2.95 vs. Myo9a^+/-^ 16.75 ± 1.6; hindlimb kicks number: WT 39.8 ± 7.95 vs. Myo9a^+/-^ 30 ± 6.94) (**Figure 5C**).

**FIGURE 6:**
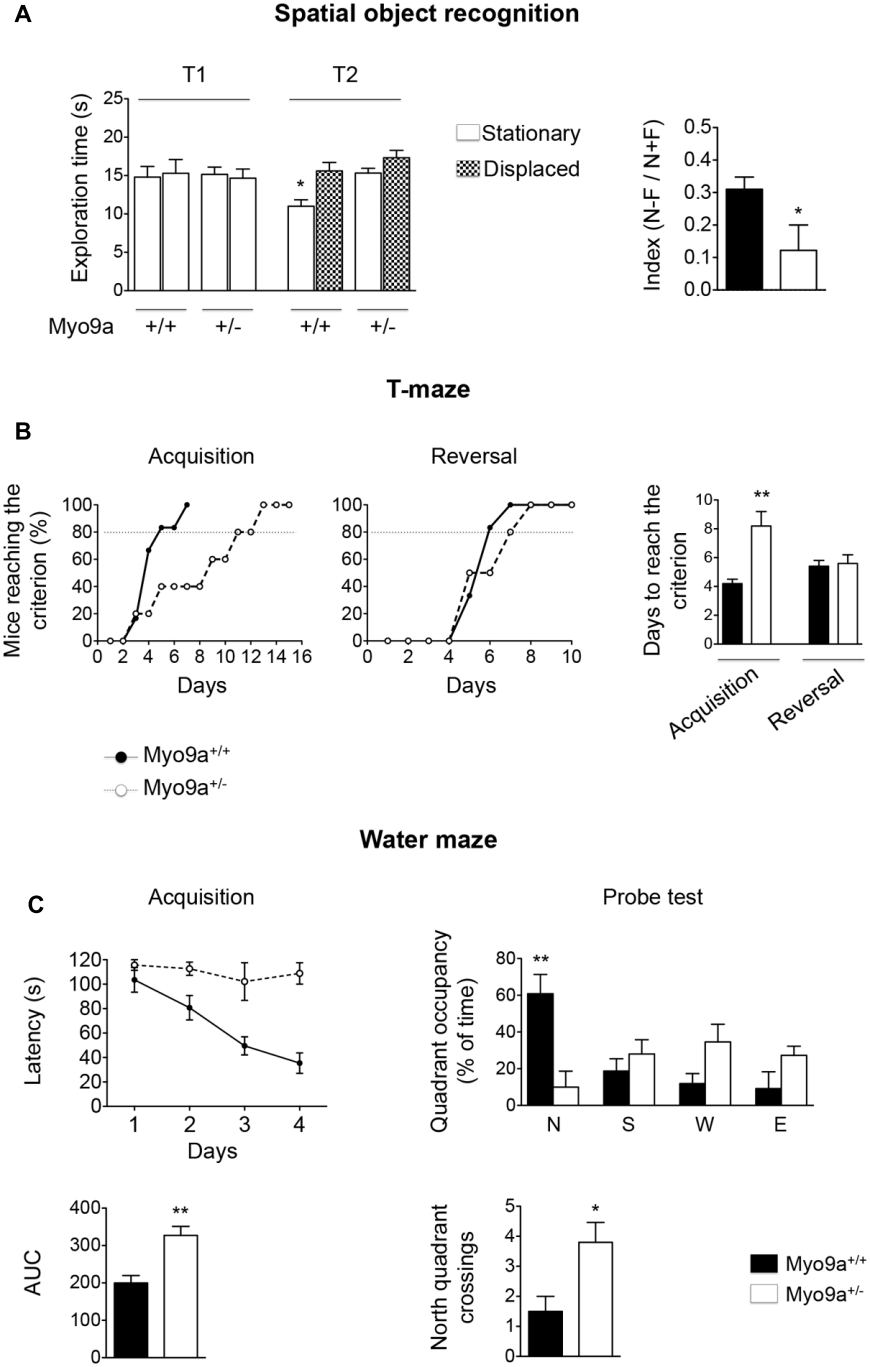
**Myo9a haploinsufficiency impaired spatial learning and memory. (A)** Spatial object recognition test. During the familiarization trial (T1), WT and Myo9a^+/-^ mice showed similar exploratory phenotypes. During the recognition trial (T2), WT mice preferentially explored the spatially displaced object over the one that remained stationary, whereas Myo9a^+/-^ mice used the same amount of time to explore the two (left). A two-way ANOVA revealed a significant difference in the genotype [*F*(1.72) = 4.05, ^∗^*p* = 0.04] but not in the location object factor [*F*(3.72) = 2.29] and interaction genotype × location object [*F*(3.72) = 1.83]. As a result, the discrimination index [(N–F)/(N+F)] was significantly reduced in Myo9a^+/-^ mice when compared to WT mice (right). **(B)** *T*-maze test. WT mice performed better during the acquisition phase when compared to Myo9a^+/-^ mice, which required more days to reach the criterion (80% of the correct choices for three days). Conversely, no significant difference was detected in the reversal phase. **(C)** Morris water maze test. Myo9a^+/-^ mice showed an increased latency to find the platform during the acquisition phase (top left). The Area Under the Curve (AUC) relative to the acquisition phase is shown (bottom left). During the probe test (when the platform was removed on the 5th day), WT mice spent significantly more time in the target zone (north quadrant, N), while Myo9a^+/-^ mice spent the same amount of time in each quadrant (top right). Furthermore, Myo9a^+/-^ mice crossed the north quadrant more times compared to WT littermates (bottom right).

Next, we performed three different tests, namely, the spatial object recognition, the *T*-maze and the Morris water maze (**Figure 6**).

In the spatial object task, the hippocampus has been proposed to combine spatial/contextual information with specific object information that is processed by the cortex (Winters et al., 2008). During the familiarization trial (T1), WT and Myo9a^+/-^ mice showed similar exploratory phenotypes as they explored the objects for the same amount of time. However, during the recognition trial (T2), WT mice preferentially explored the spatially displaced object compared to the object that remained stationary, whereas Myo9a^+/-^ mice used the same amount of time to explore the two objects. The equal amount of time spent by Myo9a^+/-^ mice to explore spatially displaced and stationary objects indicated that they failed to discriminate between the two objects (Discrimination index [(N–F)/(N+F)]: WT 0.31 ± 0.03 vs. Myo9a^+/-^ 0.122 ± 0.07, ^∗^*p* = 0.03, **Figure 6A**).

To strengthen these results, we performed *T*-maze and water maze tests, which rely on an intact hippocampus-dependent spatial memory. In the *T*-maze task, Myo9a^+/-^ mice required a longer training duration to reach the criterion (80% of correct choices for three days) during acquisition (WT 4.2 ± 0.3 vs. Myo9a^+/-^ 8.2 ± 1, *t*(8) = 3.83, ^∗∗^*p* = 0.005). Conversely, no significant difference was detected in the reversal phase (WT 5.4 ± 0.4 vs. Myo9a^+/-^ 5.6 ± 0.6, *t*(8) = 0.27) (**Figure 6B**). Similarly, in the Morris water maze, Myo9a^+/-^ mice showed an increased latency in finding the platform in the acquisition phase (Area Under the Curve, AUC: WT 199.8 ± 20.1 vs. Myo9a^+/-^ 327 ± 24.2, *t*(8) = 4.04, ^∗∗^*p* = 0.004) (**Figure 6C**, left). During the probe test Myo9a^+/-^ mice spent the same amount of time in each quadrant, while WT mice spent more time in the target zone (north quadrant) (north quadrant, N: WT 60.88 ± 10.47 vs. Myo9a^+/-^ 10.1 ± 8.657; south quadrant, S: WT 18.75 ± 6.718 vs. Myo9a^+/-^ 28 ± 7.808; west quadrant, W: WT 11.88 ± 5.473 vs. Myo9a^+/-^ 34.6 ± 9.607; east quadrant, E: WT 9.17 ± 9.175 vs. Myo9a^+/-^ 27.3 ± 5; ^∗∗^*p* < 0.01). Furthermore, Myo9a^+/-^ mice crossed more times the north quadrant compared to WT littermates (number of north quadrant crossings: WT 1.5 ± 0.5 vs. Myo9a^+/-^ 3.8 ± 0.66, ^∗^*p* = 0.03) (**Figure 6C**, right), suggesting that Myo9a^+/-^ mice did not remember the platform position and passed by the target zone rapidly.

When considered together, these data indicate that hippocampal-dependent cognitive processes are impaired upon Myo9a heterozygous deletion.

## Discussion

In the present study, we showed that Myo9a plays a role in hippocampal synapses and cognitive behavior. Myo9a is both an actin-dependent motor protein and a Rho-GAP that can inactivate RhoA (Chieregatti et al., 1998). We found that Myo9a localizes at synapses and directly interacts with the AMPAR subunit GluA2.

*In vivo*, Myo9a haploinsufficiency induced structural and molecular changes in the synapses of the CA1 area of the hippocampus. Myo9a^+/-^ mice developed thicker PSDs and an altered expression of postsynaptic markers. In particular, the major scaffolding protein of excitatory synapses, PSD95, and the newly identified Myo9a binding partner, GluA2, showed increases in their level of expression.

GluA2 is detectable in the vast majority of AMPARs and is associated with either GluA3 or GluA1 (Wenthold et al., 1996). We found that GluA2 enrichment also occurs at the plasma membrane, where both the GluA2/3 and GluA1 subunit levels increase. As expected, following an increase in the level of AMPAR at the cell surface, the amplitude and frequency of the mEPSCs were increased. Conversely, no changes in decay time or area were observed, consistent with an unaltered AMPAR subunit composition. Notably, we found no alterations in the spine number, presynaptic structure and composition, or the PPR that was used to measure presynaptic release. Altogether, these observations strongly suggest that both the amplitude and frequency of mEPSCs reflect postsynaptic AMPAR enrichment (Stein et al., 2003) and highlight a Myo9a-specific role in determining the molecular structure of the PSD.

Interestingly, LTP in hippocampal slices from Myo9a^+/-^ mice was reduced. It is reasonable to hypothesize that LTP is occluded in Myo9a^+/-^ synapses, as occurred following PSD95 overexpression, which mimics LTP (Stein et al., 2003). Indeed, PSD thickening, PSD95 recruitment and AMPAR surface enrichment are key features of potentiated synapses (Stein et al., 2003).

As a likely consequence of impaired LTP, Myo9a^+/-^ mice demonstrated behavioral deficits in hippocampus-dependent learning and memory tasks. Notably, in the *T*-maze test, Myo9a^+/-^ mice were impaired during the acquisition phase but not during the reversal-learning phase, which suggests that areas beyond the hippocampus (i.e., prefrontal cortex) are involved (Malá et al., 2015).

These observations were made in Myo9a haploinsufficient mice. Therefore, Myo9a most likely functions as a negative regulator of PSD95 and AMPAR surface expression by virtue of its motor function, Rho-GAP activity or both.

Myo9a down-regulation is expected to result in the up-regulation of RhoA activity. RhoA is normally activated following LTP induction (Murakoshi et al., 2011), and similar to PSD95 expression, constitutively active RhoA mimics and occludes LTP by increasing the mEPSCs’ amplitude and frequency (Wang et al., 2005). Furthermore, RhoA is required for both the early and late phases of LTP. During the early phase, RhoA regulates the formation of synaptic protein clusters (Wang et al., 2005; Murakoshi et al., 2011).

Because it has been recently proposed that Myo9a has a role in endocytosis via RhoA/ROCK signaling in kidney cells (Thelen et al., 2015), Myo9a might have a similar function in neurons. Indeed, the deletion of Rho-GAP OPHN1 reduces AMPAR endocytosis as a consequence of RhoA/ROCK pathway up-regulation (Khelfaoui et al., 2009). Myo9a might regulate AMPAR endocytosis and possibly AMPAR trafficking via motor protein activity through its binding to GluA2, which is the subunit that drives receptor internalization (Passafaro et al., 2001).

Even if the mechanisms remain to be elucidated, our data support a model by which Myo9a is required for synapse structure and function. It is tempting to speculate that Myo9a acts as an LTP brake that prevents synapses from undergoing uncontrolled strengthening, which is detrimental for learning and memory.

## Author Contributions

AF, LM, MF, MS, and SB designed the experiments. AF, LM, EV, LP, FL, EM, LG, and JZ performed the experiments. AF, LM, EV, EM, and DB analyzed the data. AF, LM, MF, MS, and SB wrote the paper. FP produced and validated anti GluA2/3 antibodies. MB provided essential Myo9a reagents including knockout mice, plasmids, and antibodies. All of the authors were involved in revising the manuscript for important intellectual content and gave final approval of the version to be published.

## Conflict of Interest Statement

The authors declare that the research was conducted in the absence of any commercial or financial relationships that could be construed as a potential conflict of interest.

## References

[B1] Abouhamed M. Grobe K. San I. V. Thelen S. Honnert U. Balda M. S. (2009). Myosin IXa regulates epithelial differentiation and its deficiency results in hydrocephalus. *Mol. Biol. Cell* 20 5074–5085. 10.1091/mbc.E09-04-0291 19828736 PMC2793285

[B2] Bassani S. Cingolani L. A. Valnegri P. Folci A. Zapata J. Gianfelice A. (2012). The X-linked intellectual disability protein TSPAN7 regulates excitatory synapse development and AMPAR trafficking. *Neuron* 73 1143–1158. 10.1016/j.neuron.2012.01.021 22445342 PMC3314997

[B3] Boudreau A. C. Milovanovic M. Conrad K. L. Nelson C. Ferrario C. R. Wolf M. E. (2012). A protein cross-linking assay for measuring cell surface expression of glutamate receptor subunits in the rodent brain after in vivo treatments. *Curr. Protoc. Neurosci.* Chap. 5 Unit 5.30 1–5.30.19. 10.1002/0471142301.ns0530s59 PMC3356776 22470150

[B4] Bredt D. S. Nicoll R. A. (2003). AMPA receptor trafficking at excitatory synapses. *Neuron* 40 361–379. 10.1016/S0896-6273(03)00640-8 14556714

[B5] Chieregatti E. Gärtner A. Stöﬄer H. E. Bähler M. (1998). Myr 7 is a novel myosin IX-RhoGAP expressed in rat brain. *J. Cell Sci.* 111(Pt 24), 3597–3608. 9819351 10.1242/jcs.111.24.3597

[B6] Correia S. S. Bassani S. Brown T. C. Lisé M. F. Backos D. S. El-Husseini A. (2008). Motor protein-dependent transport of AMPA receptors into spines during long-term potentiation. *Nat. Neurosci.* 11 457–466. 10.1038/nn2063 18311135

[B7] DeFelipe J. Marco P. Busturia I. Merchán-Pérez A. (1999). Estimation of the number of synapses in tqhe cerebral cortex: methodological considerations. *Cereb. Cortex* 9 722–732. 10.1093/cercor/9.7.722 10554995

[B8] Dosemeci A. Tao-Cheng J. H. Vinade L. Winters C. A. Pozzo-Miller L. Reese T. S. (2001). Glutamate-induced transient modification of the postsynaptic density. *Proc. Natl. Acad. Sci. U.S.A.* 98 10428–10432. 10.1073/pnas.181336998 11517322 PMC56977

[B9] Ferri A. L. Cavallaro M. Braida D. Di Cristofano A. Canta A. Vezzani A. (2004). Sox2 deficiency causes neurodegeneration and impaired neurogenesis in the adult mouse brain. *Development* 131 3805–3819. 10.1242/dev.01204 15240551

[B10] Gorman S. W. Haider N. B. Grieshammer U. Swiderski R. E. Kim E. Welch J. W. (1999). The cloning and developmental expression of unconventional myosin IXA (MYO9A) a gene in the Bardet-Biedl syndrome (BBS4) region at chromosome 15q22-q23. *Genomics* 59 150–160. 10.1006/geno.1999.5867 10409426

[B11] Hanley P. J. Xu Y. Kronlage M. Grobe K. Schön P. Song J. (2010). Motorized RhoGAP myosin IXb (Myo9b) controls cell shape and motility. *Proc. Natl. Acad. Sci. U.S.A.* 107 12145–12150. 10.1073/pnas.0911986107 20566876 PMC2901435

[B12] Huganir R. L. Nicoll R. A. (2013). AMPARs and synaptic plasticity: the last 25 years. *Neuron* 80 704–717. 10.1016/j.neuron.2013.10.025 24183021 PMC4195488

[B13] Kenney J. W. Adoff M. D. Wilkinson D. S. Gould T. J. (2011). The effects of acute, chronic, and withdrawal from chronic nicotine on novel and spatial object recognition in male C57BL/6J mice. *Psychopharmacology (Berl.)* 217 353–365. 10.1007/s00213-011-2283-7 21487656 PMC3161157

[B14] Kessels H. W. Malinow R. (2009). Synaptic AMPA receptor plasticity and behavior. *Neuron* 61 340–350. 10.1016/j.neuron.2009.01.015 19217372 PMC3917551

[B15] Khelfaoui M. Pavlowsky A. Powell A. D. Valnegri P. Cheong K. W. Blandin Y. (2009). Inhibition of RhoA pathway rescues the endocytosis defects in Oligophrenin1 mouse model of mental retardation. *Hum. Mol. Genet.* 18 2575–2583. 10.1093/hmg/ddp189 19401298 PMC2701329

[B16] Malá H. Andersen L. G. Christensen R. F. Felbinger A. Hagstrøm J. Meder D. (2015). Prefrontal cortex and hippocampus in behavioural flexibility and posttraumatic functional recovery: reversal learning and set-shifting in rats. *Brain Res. Bull.* 116 34–44. 10.1016/j.brainresbull.2015.05.006 26033702

[B17] Morris R. G. Garrud P. Rawlins J. N. O’Keefe J. (1982). Place navigation impaired in rats with hippocampal lesions. *Nature* 297 681–683. 10.1038/297681a0 7088155

[B18] Murakoshi H. Wang H. Yasuda R. (2011). Local, persistent activation of Rho GTPases during plasticity of single dendritic spines. *Nature* 472 100–104. 10.1038/nature09823 21423166 PMC3105377

[B19] Passafaro M. Piëch V. Sheng M. (2001). Subunit-specific temporal and spatial patterns of AMPA receptor exocytosis in hippocampal neurons. *Nat. Neurosci.* 4 917–926. 10.1038/nn0901-917 11528423

[B20] Perry T. A. Torres E. M. Czech C. Beyreuther K. Richards S. Dunnett S. B. (1995). Cognitive and motor function in transgenic mice carrying excess copies of the 695 and 751 amino acid isoforms of the amyloid precursor protein gene. *Alzheimers Res.* 1 5–14.

[B21] Pitsikas N. Rigamonti A. E. Cella S. G. Locatelli V. Sala M. Muller E. E. (2001). Effects of molsidomine on scopolamine-induced amnesia and hypermotility in the rat. *Eur. J. Pharmacol.* 426 193–200. 10.1016/S0014-2999(01)01164-5 11527544

[B22] Reinhard J. Scheel A. A. Diekmann D. Hall A. Ruppert C. Bähler M. (1995). A novel type of myosin implicated in signalling by rho family GTPases. *EMBO J.* 14 697–704. 7882973 10.1002/j.1460-2075.1995.tb07048.x PMC398134

[B23] Sala M. Braida D. Lentini D. Busnelli M. Bulgheroni E. Capurro V. (2011). Pharmacologic rescue of impaired cognitive flexibility, social deficits, increased aggression, and seizure susceptibility in oxytocin receptor null mice: a neurobehavioral model of autism. *Biol. Psychiatry* 69 875–882. 10.1016/j.biopsych.2010.12.022 21306704

[B24] Shi S. Hayashi Y. Esteban J. A. Malinow R. (2001). Subunit-specific rules governing AMPA receptor trafficking to synapses in hippocampal pyramidal neurons. *Cell* 105 331–343. 10.1016/S0092-8674(01)00321-X 11348590

[B25] Stein V. House D. R. Bredt D. S. Nicoll R. A. (2003). Postsynaptic density-95 mimics and occludes hippocampal long-term potentiation and enhances long-term depression. *J. Neurosci.* 23 5503–5506. 12843250 10.1523/JNEUROSCI.23-13-05503.2003 PMC6741246

[B26] Takumi Y. Ramírez-León V. Laake P. Rinvik E. Ottersen O. P. (1999). Different modes of expression of AMPA and NMDA receptors in hippocampal synapses. *Nat. Neurosci.* 2 618–624. 10.1038/10172 10409387

[B27] Talani G. Biggio G. Sanna E. (2011). Enhanced sensitivity to ethanol-induced inhibition of LTP in CA1 pyramidal neurons of socially isolated C57BL/6J mice: role of neurosteroids. *Front. Endocrinol. (Lausanne)* 2:56 10.3389/fendo.2011.00056 PMC3355925 22649377

[B28] Teyler T. J. Discenna P. (1984). Long-term potentiation as a candidate mnemonic device. *Brain Res.* 319 15–28. 10.1016/0165-0173(84)90027-4 6324959

[B29] Thelen S. Abouhamed M. Ciarimboli G. Edemir B. Bähler M. (2015). The Rho GAP Myosin IXa is a regulator of kidney tubule function. *Am. J. Physiol. Renal Physiol.* 309 F501–F513. 10.1152/ajprenal.00220.2014 26136556

[B30] Van’t Veer A. Bechtholt A. J. Onvani S. Potter D. Wang Y. Liu-Chen L. Y. (2013). Ablation of kappa-opioid receptors from brain dopamine neurons has anxiolytic-like effects and enhances cocaine-induced plasticity. *Neuropsychopharmacology* 38 1585–1597. 10.1038/npp.2013.58 23446450 PMC3682153

[B31] Wang H. G. Lu F. M. Jin I. Udo H. Kandel E. R. de Vente J. (2005). Presynaptic and postsynaptic roles of NO, cGK, and RhoA in long-lasting potentiation and aggregation of synaptic proteins. *Neuron* 45 389–403. 10.1016/j.neuron.2005.01.011 15694326

[B32] Wenthold R. J. Petralia R. S. Blahos J. I. I. Niedzielski A. S. (1996). Evidence for multiple AMPA receptor complexes in hippocampal CA1/CA2 neurons. *J. Neurosci.* 16 1982–1989. 8604042 10.1523/JNEUROSCI.16-06-01982.1996 PMC6578515

[B33] Winters B. D. Saksida L. M. Bussey T. J. (2008). Object recognition memory: neurobiological mechanisms of encoding, consolidation and retrieval. *Neurosci. Biobehav. Rev.* 32 1055–1070. 10.1016/j.neubiorev.2008.04.004 18499253

[B34] Wyszynski M. Kim E. Yang F. C. Sheng M. (1998). Biochemical and immunocytochemical characterization of GRIP, a putative AMPA receptor anchoring protein, in rat brain. *Neuropharmacology* 37 1335–1344. 10.1016/S0028-3908(98)00120-8 9849669

